# Parallel-META 3: Comprehensive taxonomical and functional analysis platform for efficient comparison of microbial communities

**DOI:** 10.1038/srep40371

**Published:** 2017-01-12

**Authors:** Gongchao Jing, Zheng Sun, Honglei Wang, Yanhai Gong, Shi Huang, Kang Ning, Jian Xu, Xiaoquan Su

**Affiliations:** 1 Single-Cell Center, Shandong Key Laboratory of Energy Genetics and CAS Key Laboratory of Biofuels, Qingdao Institute of Bioenergy and Bioprocess Technology, Chinese Academy of Sciences. Qingdao, Shandong, 266101, China

## Abstract

The number of metagenomes is increasing rapidly. However, current methods for metagenomic analysis are limited by their capability for in-depth data mining among a large number of microbiome each of which carries a complex community structure. Moreover, the complexity of configuring and operating computational pipeline also hinders efficient data processing for the end users. In this work we introduce Parallel-META 3, a comprehensive and fully automatic computational toolkit for rapid data mining among metagenomic datasets, with advanced features including 16S rRNA extraction for shotgun sequences, 16S rRNA copy number calibration, 16S rRNA based functional prediction, diversity statistics, bio-marker selection, interaction network construction, vector-graph-based visualization and parallel computing. Application of Parallel-META 3 on 5,337 samples with 1,117,555,208 sequences from diverse studies and platforms showed it could produce similar results as QIIME and PICRUSt with much faster speed and lower memory usage, which demonstrates its ability to unravel the taxonomical and functional dynamics patterns across large datasets and elucidate ecological links between microbiome and the environment. Parallel-META 3 is implemented in C/C++ and R, and integrated into an executive package for rapid installation and easy access under Linux and Mac OS X. Both binary and source code packages are available at http://bioinfo.single-cell.cn/parallel-meta.html.

With the rapid development of Next Generation Sequencing technologies, metagenome datasets have been increasing explosively, both in sample number and in sequence volume. Data mining across hundreds or even thousands of metagenomes promises to uncover highly valuable biological information, such as a landscape view of microbiota structure and function^1^, or the fine association between microbiota dynamics and human health status^2^ or environmental factors^3^. Such integrated and in-depth comparison of taxonomical structure or functional profile in large-scale metagenomics datasets has become important or even essential in the many microbiota-enabled applications^4^.

A number of methods have been developed for metagenomic analysis. MetaPhlAn^5^ profiles microbial community composition using a universal biomarker gene, yet it was designed for only shotgun metagenome datasets, and lacks in-depth analysis proceedings such as quantitative similarity calculation and diversity evaluation among multiple samples. Mothur^6^ and QIIME^7^ are widely used toolkits for analysing 16S rRNA amplicon based metagenome datasets, however their computing throughput has become a bottleneck; moreover they both require dozens of dependency packages, thus the installation, configuration and operation are tedious and complicated.

Here we propose Parallel-META 3, a comprehensive and automatic software package (Fig. 1) that provides rapid data mining on taxonomy and metabolic function across a large number of metagenome datasets. Compared to previous versions^8^,^9^, its advanced features include 16S rRNA copy number calibration, 16S rRNA based functional prediction, diversity statistics, biomarkers selection, and data visualization based on high-quality vector graphs. For high-performance computing over massive datasets, all processing steps in Parallel-META 3 are implemented using C/C++ and/or R with parallel computing techniques and self-adapted load balancing strategy. In addition, this software is encapsulated and integrated into a well-configured and full-automatic pipeline for easy installation and friendly user experience. Tests on 5,337 samples with 1,117,555,208 16S amplicon sequences showed that Parallel-META 3 is able to uncover dynamics of microbiomic features and patterns in both taxonomical and functional aspects with higher efficiency, optimized memory usage and uncompromised precision from large-scale metagenome datasets.

## Results

To evaluate the performance of Parallel-META 3, we prepared four test datasets collected from different studies and produced by different platforms (Table 1). Dataset 1 and 2 contained 4,799 16S rRNA amplicon samples in total from healthy adults at five body sites (nasal, gastrointestinal, oral, skin and urogenital) produced by Human Microbiome Project^10^. Dataset 3 contained 528 human gut 16S rRNA amplicon samples from healthy adults, children less than 3 years old (C-3) and children of 3–17 years old (C-17) from three countries (Malawi, USA and Venezuela) produced by Yatsunenko, *et al*.^11^ These three datasets were employed as representatives of 16S rRNA amplicon based microbiome data.

On the other hand, in order to test the performance on shotgun metagenomics data, Datasets 4 was included, which contained 10 artificial metagenomic shotgun samples that were simulated with nine organisms using Dwgsim^12^ (version 0.1.8). These 10 samples were divided into two groups based on proportion of organisms to test the sensitivity of Parallel-META 3 in distinguishing samples with different community patterns (detailed design of simulation in Table S1 of Supplementary file S1).

Results of 16S rRNA amplicon samples from the first three datasets were generated via Parallel-META 3 (version 3.3.2), then they were compared to the taxonomical structure reported by QIIME^7^ (version 1.9.0) and functional profiles predicted by PICRUSt^13^ (version 1.0) that worked the benchmark. For the artificial metagenomic shotgun sequences of Dataset 4, the taxonomy structure generated by Parallel-META 3 (version 3.3.2) was compared to the simulation design. OTUs (Operational Taxonomy Units) were picked at 97% similarity level, functions profiles were predicted based on KO (KEGG Ontology) database^14^, and other parameters were kept as the default configuration. Moreover, statistical analysis figures were automatically generated via the visualization capability of Parallel-META 3. All tests were performed on a rack server with quad Intel Xeon E5-4620 CPUs (32 physical cores, 64 threads in total) and 256GB RAM.

### Higher efficiency of Parallel-META 3 in computing time and memory usage than the benchmark

**Figure 2:**
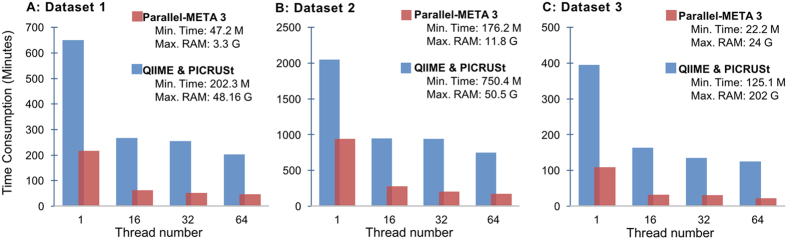
The running time and memory consumption of Parallel-META 3 compared to the benchmark software of QIIME and PICRUSt with 16S rRNA amplicon datasets.

We illustrated the efficiency advantages of Parallel-META 3 by comparing the running time and memory usage of all analysis processes with the three 16S rRNA amplicon datasets to those of the benchmark by QIIME and PICRUSt. All tool packages were invocated in parallel computing model using 1, 16, 32 and 64 threads, respectively. Benefited by the whole-process parallel scheduling, multi-thread memory sharing and C++ programming, Parallel-META 3 achieved a ~5× faster speed (Fig. 2) than the benchmark scores, yet with remarkably lower RAM usage on identical hardware configurations, such as for Dataset 1 the benchmark software used over 48 GB but Parallel-META 3 only took 3.3 GB. In addition, the speedup ratio was independent of either the source or the sequence type of the input samples. This acceleration demonstrated the capability of Parallel-META 3 on rapid evaluation of input samples, which is essential for in-depth data mining with massive amount of samples and complex community structures.

### Identification of microbiota structure pattern from 16S rRNA amplicon samples

**Figure 3:**
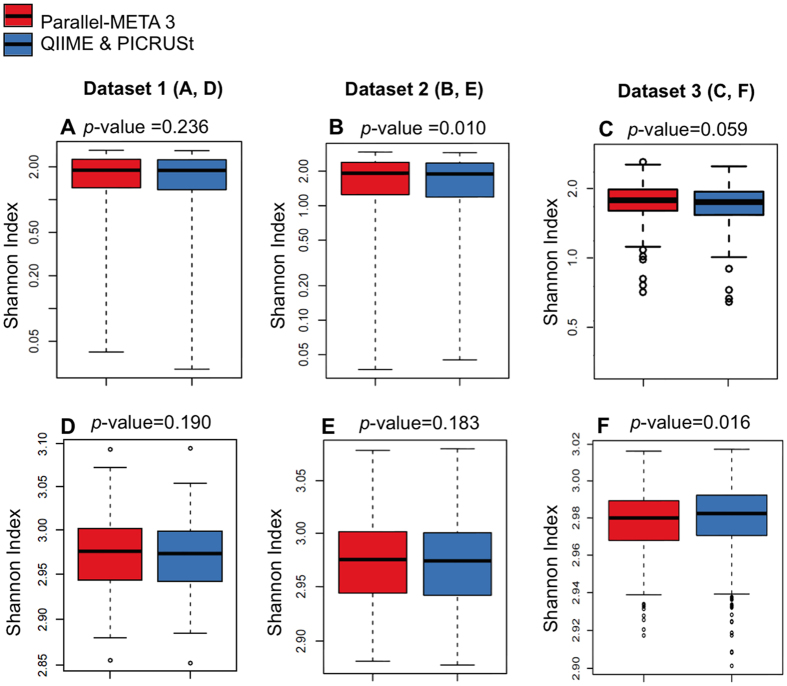
Shannon index α diversity of 16S rRNA amplicon datasets validated by QIIME at the taxonomical genus level (**A**): Dataset 1, (**B**): Dataset 2 and (**C**): Dataset (3) and by PICRUSt at the functional pathway level (**D**): Dataset 1, (**E**): Dataset 2 and (**F**): Dataset (3). Simpson index results were shown in Figure S1 in Supplementary file S1.

**Figure 4:**
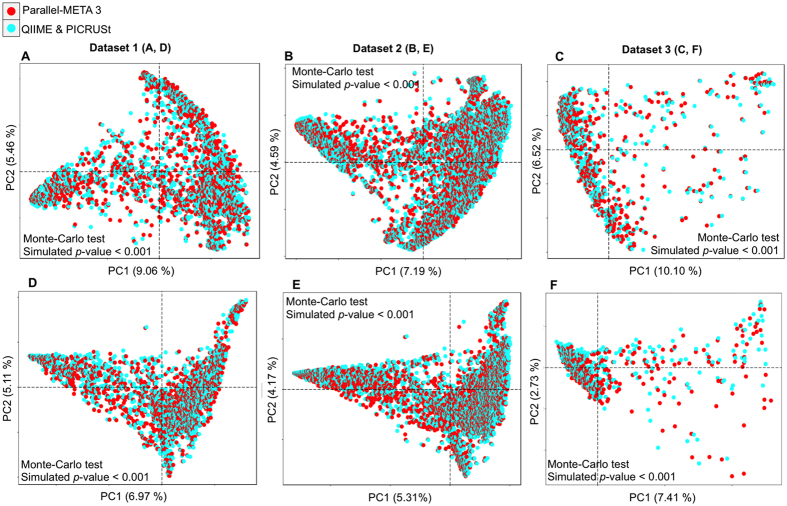
The β diversity patterns of 16S rRNA amplicon datasets based on Meta-Storms distances validated by QIIME (**A**): Dataset 1, (**B**): Dataset 2 and (**C**): Dataset (3) and by PICRUSt (**D**): Dataset 1, (**E**): Dataset 2 and (**F**): Dataset (3).

**Figure 5:**
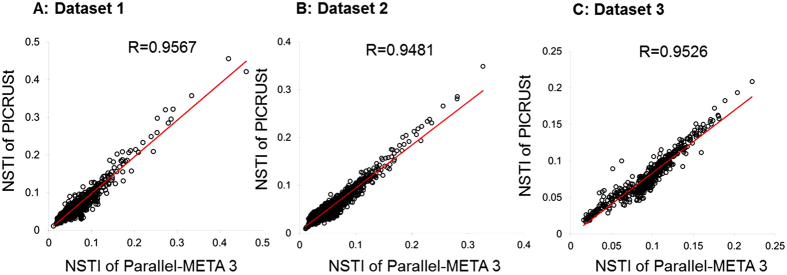
Correlations of NTSI values calculated respectively by Parallel-META 3 and PICRUSt on 16S rRNA amplicon datasets.

Community structure is the basis for in-depth interpretation of microbiota. We verified the overall taxonomical patterns and predicted metabolic profiles of three 16S rRNA amplicon datasets generated by Parallel-META 3 with the benchmark from both α diversity and β diversity aspects. The α diversity was calculated by Shannon index and Simpson index at the genus level and the KEGG pathway level; the β diversity was examined based on pair-wised Meta-Storms^15^ distance of OTUs and KOs. Since the 16S rRNA copy number calibration (refer to Methods section for details) was not supported in QIIME, we disabled this option in taxonomy analysis of Parallel-META 3 for consensus purpose. No significant difference was observed between the community structures reported by Parallel-META 3 and the benchmark on α diversity (Wilcoxon rank-sum test; *p*-value > 0.01; Fig. 3). Moreover, the two methods generated similar β diversity patterns in PCoA as verified by Monte-Carlo test (10,000 permutations, *p*-value < 0.001; Fig. 4). Furthermore, the reliability of metabolic prediction was examined by NSTI (Nearest Sequenced Taxa Index) values of PICRUSt^13^. The strong Pearson correlation (R > 0.9) also confirmed the uncompromised precision in functional profiling for Parallel-META 3 (Fig. 5).

### Interpreting the diversity of 16S rRNA amplicon samples

**Figure 6:**
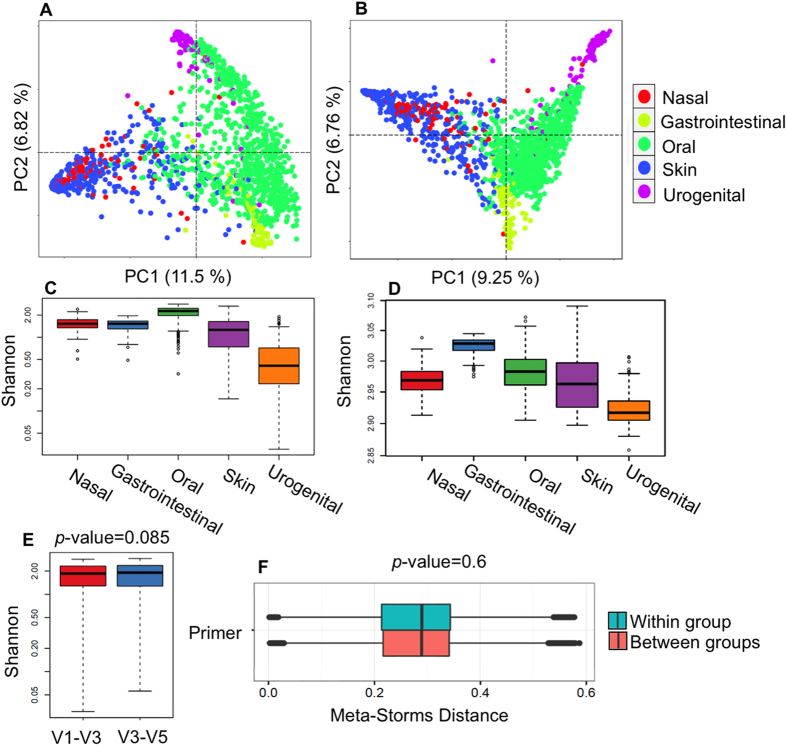
Community diversity variations among body sites. (**A** and **B**): Partition of β diversity on taxonomy and functions based on Meta-Storms distances of Dataset 1; (**C** and **D**): Shannon index α diversity of Dataset 1 on genus level and pathway level; (**E** and **F**): Comparison of 16S rRNA reads from V1-V3 region (Dataset 1) and V3-V5 region (Dataset 2) on taxonomical α diversity and β diversity. Diversity analysis of Dataset 2 was included in Figure S2 of supplementary file S1.

**Figure 7:**
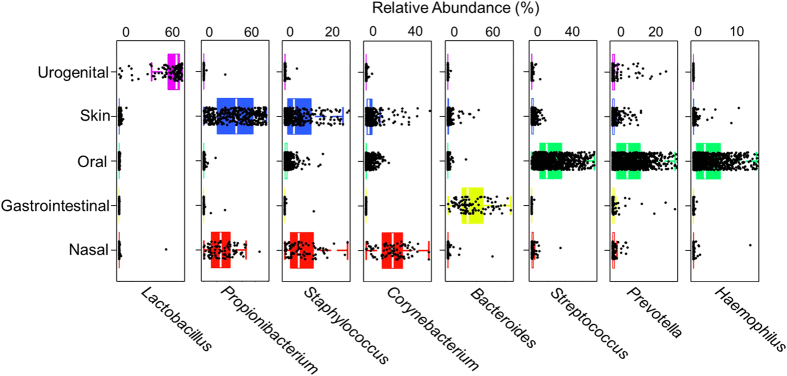
The most abundant genera of each of the body sites as identified by biomarker analysis.

Since change in microbial diversity in microbiota samples has been linked to several human diseases, we assessed the capability of Parallel-META 3 in α diversity and β diversity statistics via both taxonomical structure and predicted functional profiles. For human-associated microbiota samples from Dataset 1 and Dataset 2, Parallel-META 3 differentiated samples from each body site via β diversity by their taxonomical and functional profiles (Fig. 6A and B). Moreover, the distinct degree of microbiomic complexity in each body site was revealed via α diversity (Fig. 6C and D, Wilcoxon rank-sum test *p*-value < 0.01): oral communities feature especially diverse taxonomical membership, vaginal sites harbor particularly simple communities, while gut samples had the most complex metabolic functions. This significance was also confirmed by previous works of The Human Microbiome Project Consortium, *Nature*^16^. Furthermore, biomarker analysis at the genus level suggested that each body site could be characterized and distinguished by the relative abundance of just a few taxa (Fig. 7): for example, skin communities were specifically dominated by *Propionibacterium* and *Staphylococcus*, while oral samples contained abundant *Streptococcus* and *Prevotella*. In addition, it was apparent that whether 16S rRNA reads were from V1-V3 and V3-V5 regions did not affect the diversity analysis results (Fig. 6E and F).

**Figure 8:**
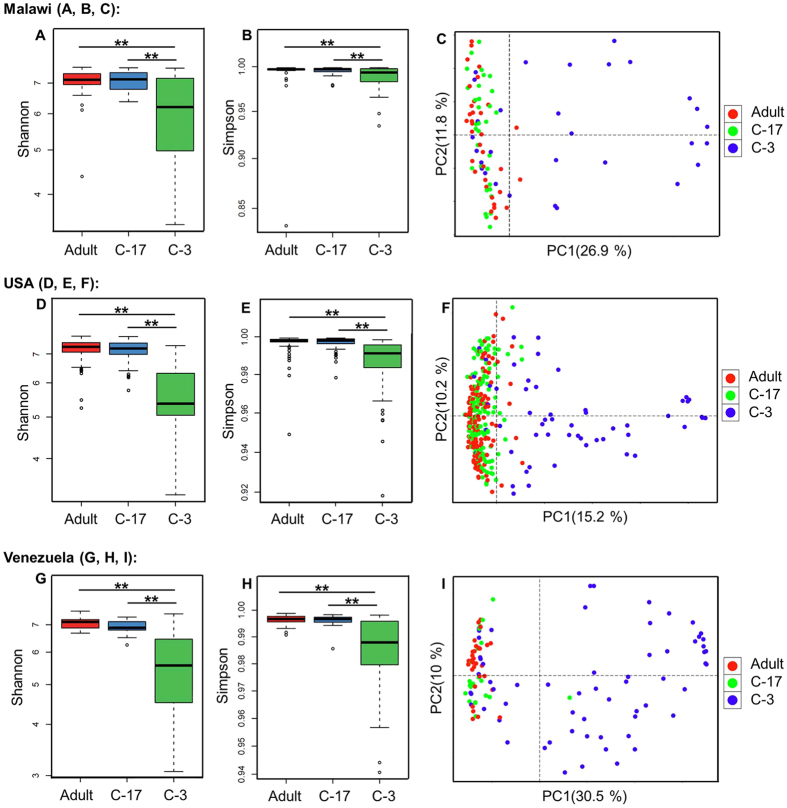
Taxonomy diversity among microbiota samples of adults and children from three countries. α diversity was measured by Shannon index and Simpson index (**A** and **B**) for Malawi, (**D** and **E**) for US, (**G** and **H**) for Venezuela); β diversity was illustrated in PCoA based on Meta-Storms distance (**C**) for Malawi, (**F**) for US and (**I**) for Venezuela). Refer to Figure S3 in Supplementary file S1 for diversity of functional profiles.

**Figure 9:**
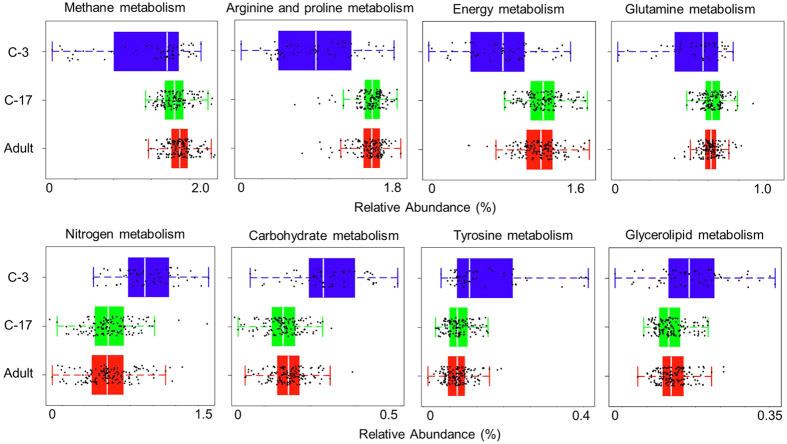
Functional pathways that are differentially distributed between adults and children microbiome.

**Figure 10:**
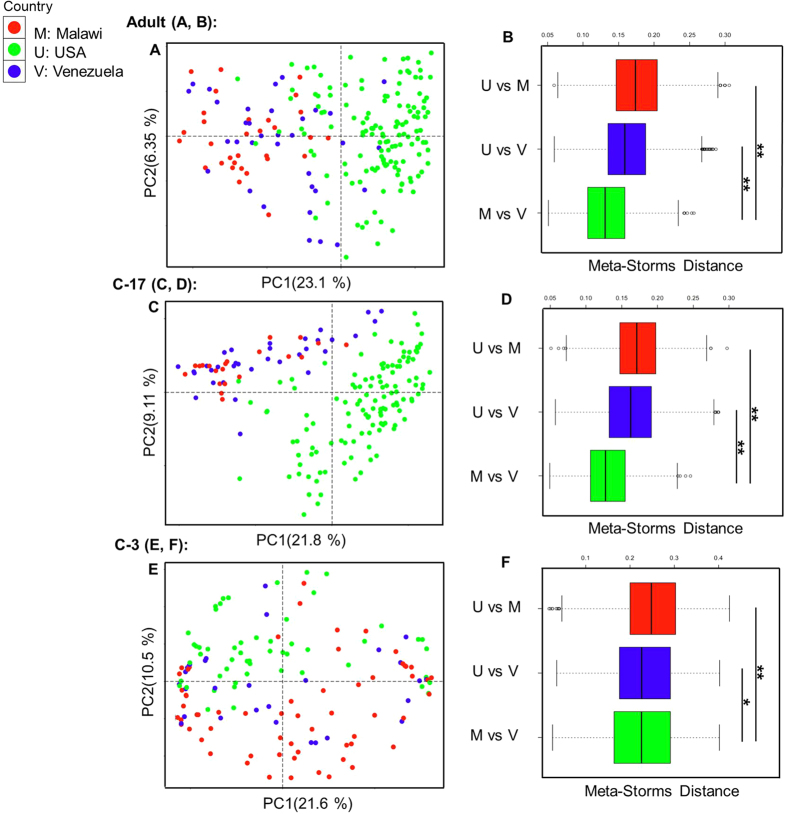
Significant taxonomical variation of microbial community compositions was observed among gut samples from different countries of all age stages. Meta-Storms distances between the Malawi samples and the Venezuela samples were smaller than that between other pairs. Functional profiles exhibited a similar pattern (refer to Figure S4 in Supplementary file S1 for details).

Using the human gut microbiome samples of Dataset 3, Parallel-META 3 generated results that described the global patterns of age-associated changes and partitions among populations and countries. In this case we observed that gut samples from children under three-year old had a much lower α diversity, and interpersonal variation as evaluated by β diversity on both taxonomical and functional levels was significantly greater among children under three-year old than among 3-7-year old children or adults; such a pattern was robust to change in geography (Fig. 8). On the other hand, functional analysis uncovered the specific pathways that discriminated the microbiomes of different ages (Fig. 9): methane, arginine, proline, energy and glutamine metabolisms were higher in the adult microbiomes, while nitrogen, carbohydrate, tyrosine and glycerolipid metabolisms were enriched in baby microbiomes. In addition, notable differences in functional profile of microbiota were found among individuals that lived in different countries, with especially pronounced separation observed between the Malawian & Venezuelans and the US gut communities across all age stages (Fig. 10).

### Identification of microbiota structure pattern by simulated metagenomic shotgun sequences

**Figure 11:**
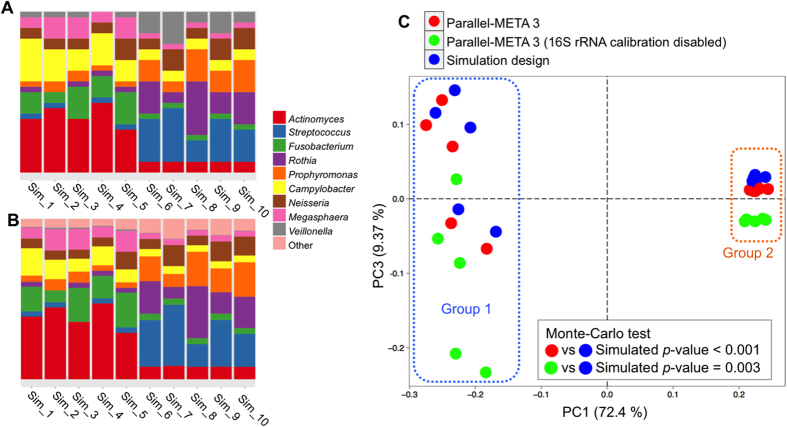
Community structure patterns parsed from metagenomic shotgun sequences of Dataset 4. (**A**) Simulation design at the genus level; (**B**). Parallel-META 3 analysis results at the genus level; (**C**). β diversity comparison via PCA among Parallel-META 3 (16S rRNA copy number calibration enabled; the default option), Parallel-META 3 (16S rRNA copy number calibration disabled) and simulation design.

Using Dataset 4, the ability of Parallel-META 3 to process shotgun metagenome data was examined by comparing the community structures reported by our software to the simulation design with 10 samples. Parallel-META 3 was used to profile shotgun sequences at the genus level, and the relative abundance of all organisms then estimated with 16S rRNA copy number calibration. Parallel-META 3 finished all processes on the 10 simulated shotgun samples in 15 minutes (892.06 s) with maximum RAM usage of 1.6 GB. The microbiota patterns were presented in bar charts (Fig. 11A and B) and then compared to the simulation design in PCA (Fig. 11C) on β diversity by Monte-Carlo test with 10,000 permutations (*p*-value < 0.001). Based on the calculated organism proportions, the 10 samples were separated into the two groups that were consistent with the simulation design, thus confirming the reliability of Parallel-META 3 in handling metagenomic shotgun sequences. In addition, had the analysis been performed without 16S rRNA copy number calibration in PCA (Fig. 11C), a noticeable deviation of the results from the true answer in the simulation design was observed (*p*-value = 0.003). This indicated that the new feature of 16S rRNA copy number calibration in Parallel-META 3 could improve the accuracy of estimating relative abundances of organisms in microbiota.

## Discussion

With the rapid expansion of microbiota research, it is becoming increasingly important to perform in-depth data mining of large microbiome datasets or compare datasets produced from different studies. However, the challenge associated with big-data computing has limited the data size-range and samples number that can be effectively analysed. On one hand, such analysis usually cannot be finished in only single round, instead requires several iterations for parameter adjustment, meta-data (sampling information) arrangement, samples insertion or removal, or result optimization; computing-intensiveness of such processes frequently causes an unacceptable amount of wait time, especially when the number of samples reaches thousands. On the other hand, hundreds of Giga-bytes of RAM consumption as well as larger amount of computing resources are usually required for such analyses. The parallel computing provided by Parallel-META 3 tackles this challenge by both increasing the running speed for multi-round configurable analysis and reducing the requirement of computing hardware.

Moreover, the complexity of hierarchical structures and dependency packages associated with current metagenome analysis pipelines can become a practical barrier for installations and operations. Targeting this issue, we have enhanced the users experience of Parallel-META 3 in both installation and access by: (*i*) encapsulating software components into a single package to allow installation with little user configurations, (*ii*) organizing all steps for automatic execution by a single command line with user-defined parameters, and (*iii*) simplifying the visualization for users via high-quality vector graphs to allow nimble and flexible interrogation of the data.

In summary, Parallel-META 3 enables high-throughput and in-depth data mining from a large number of microbial community samples with high efficiency and out-standing experience. Further development of this and related tools that tackle the challenges associated with comparison, searching and mining of large-scale and heterogeneous microbiome datasets should help to fulfil the promise of microbiome research in various scientific disciplines and application areas.

## Methods

### Taxonomic and functional profiling

Parallel-META 3 accepts both shotgun metagenomic sequences and 16S rRNA amplicon sequences. For shotgun sequences, Parallel-MEA 3 first constructs Hidden Markov Models using all bacterial 16S rRNA sequences of SILVA^17^ (version 123), and predicts the 16S rRNA gene fragments in metagenomic shotgun samples from both the forward sequences and reversed complementary sequences by HMMER^18^ (version 3.1, e-value < 1e-5). Then Parallel-META 3 extracts all the 16S rRNA fragments from metagenomic shotgun sequences for profiling. All 16S rRNA gene sequences (either extracted from shotgun sequences or 16S rRNA amplicon reads) are aligned to the Parallel-META 3 reference database by Bowtie2^19^ for OTU picking, taxonomical annotation and phylogeny construction. The reference 16S rRNA sequences are from a customized database that integrates GreenGenes^20^ (version 13-8, sequence similarity on 97% level) with RDP and SILVA consensus taxonomy annotation (assigned by BLASTN with e-value < 1e-30 and similarity >97%), which raised the proportion of annotated sequences at the genus level from 35.8% to 81.5% (refer to Figure S5 in Supplementary file S1 for details). The phylogenetic architecture of all reference sequences is built by FastTree^21^. Since 16S rRNA gene copy number varies greatly among different bacterial species, Parallel-META 3 also calculates the precise relative abundance of each organism by 16S rRNA copy number calibration using IMG database^22^. In addition, considering that the uneven sequencing depth (number of sequences) among multiple samples may introduce bias in detecting diversity patterns^23^, an optional sequence rarefaction for sequencing depth normalization at the OTU level is provided after the taxonomic profiling.

For prediction and annotation of functional profile, Parallel-META 3 re-implements the PICRUSt^13^ algorithm using KEGG database^14^ to estimate all the functional genes harboured in a microbiota using 16S rRNA gene OTUs. The functional genes are annotated by KO (KEGG Ontology) and KEGG pathway. Parallel-META 3 also measures the prediction accuracy by the NSTI (Nearest Sequenced Taxon Index) value^13^, which is calculated by the sum of distances between OTUs and their nearest individually sequenced relatives in the phylogenetic architecture.

After taxonomical and functional profiling, Parallel-META 3 parses out the sequence counts and relative abundances (normalized into 0–100%) for all OTUs, and estimates the same information for annotated taxa from the phylum level to the genus level, as well as the genes and the pathways. Such data is framed into tables that are compatible for further analysis in Parallel-META 3 and also suitable for manual examination by users.

### The α diversity evaluation and statistics

Parallel-META 3 evaluates α diversity that describes the inner complexity of each individual microbiota sample. This process generates rarefaction curves of α diversity based on observed OTU number and Shannon index to determine the adequacy of the sequencing depth. The rarefaction performs a series of random sequence selection on different sequencing depth with bootstrap (default is 20, and can also be defined by users), and the α diversity in the curves is calculated by the mean sequence count of each OTU among the bootstrapping procedures. Then the influence of each environmental factor on α diversity is quantitatively evaluated by multivariate statistical analysis with Shannon index, Simpson index and CHAO1 index.

### The β diversity evaluation and statistics

Parallel-META 3 examines β diversity of multiple microbiota samples based on their pair-wise similarity matrix to discover the patterns of organism/gene sharing and variation among samples. The quantitative similarity between each sample pair is computed by Meta-Storms^15^ algorithm, which considers both the relative abundance of OTUs existent in two samples and the distances among OTUs in the phylogenetic architecture. The β diversity evaluation includes unsupervised hierarchical clustering, supervises clustering using PCA (Principle Component Analysis), PCoA (Principle Co-ordinate Analysis) and multivariate statistical analysis that quantitatively evaluates the correlation between environmental factors and the sample similarities.

### Biomarker discovery

Parallel-META 3 can also identify key organisms or functional genes that are highly correlated with the variations of the habitats or other types of metadata. Organisms or genes with significant differences among microbial community samples were firstly chosen using Kruskal-Wallis or Wilcoxon rank-sum test as candidate makers, and these candidate makers are then ranked based on their contribution to the differentiation among samples using the Random Forest algorithm.

### Construction of microbial interaction network

The microbial interaction network is constructed to explore co-occurrence and co-exclusion patterns of organisms or functional genes across microbial community samples. In the interaction network, each node represents a single organism (or gene), and nodes are connected by links that represent their correlation coefficient of abundance variation among multiple samples^24^. Then Parallel-META 3 illustrates the global pattern among multiple samples by the network’s topological characters such as nodes number, isolated island number, density, diameter, radius and centralization (an example is provided in Figure S6 in supplementary file S1).

### Parallel computing and optimization

Parallel-META 3 was designed and constructed by parallel computing based on C/C++ OpenMP library. All process steps are allocated to independent threads and in parallel invocated in multiple CPUs or CPU cores, with a dynamic threads scheduling strategy for optimized load balancing, and shared memory spaces for lower RAM usage. The thread number is automatically assigned based on hardware detection, and can also be defined or customized by the users.

## Additional Information

**How to cite this article**: Jing, G. *et al*. Parallel-META 3: Comprehensive taxonomical and functional analysis platform for efficient comparison of microbial communities. *Sci. Rep.* **7**, 40371; doi: 10.1038/srep40371 (2017).

**Publisher's note:** Springer Nature remains neutral with regard to jurisdictional claims in published maps and institutional affiliations.

## Supplementary Material

Supplementary Information

## Figures and Tables

**Figure 1:**
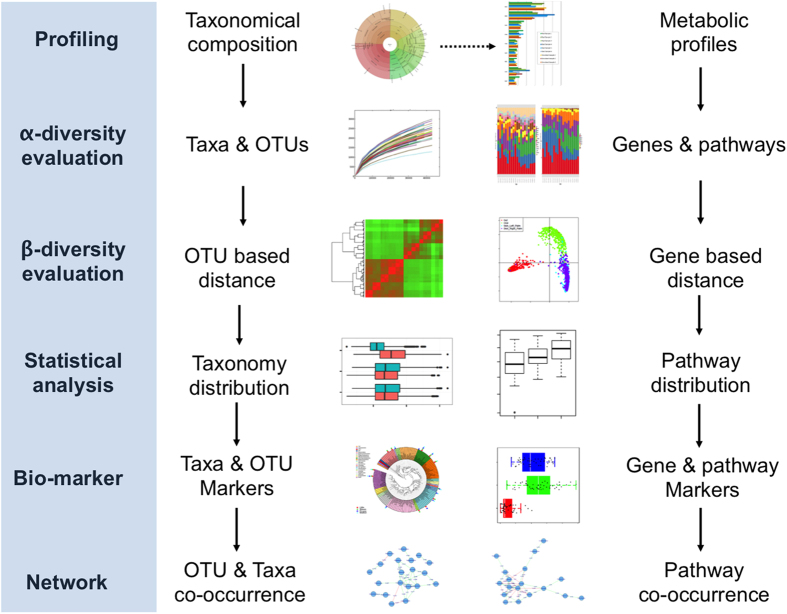
Overall workflow of Parallel-META 3. All analysis steps were implemented in C/C++ and/or R with optimized parallel computing, and well configured in to a fully automatic pipeline package.

**Table 1:** Detailed information of the test datasets.

Dataset	Sequence type	Study	# of samples	# of sequences	platform
Dataset 1	16S rRNA amplicon V1-V3 region	Human Microbiome Project	1,547	28,089,946	Roche 454 FLX
Dataset 2	16S rRNA amplicon V3-V5 region	Human Microbiome Project	3,252	42,359,290	Roche 454 FLX
Dataset 3	16S rRNA amplicon V4 region	Yatsunenko, *et al., Nature*, 2012	528	1,093,740,363	Illumina HiSeq 2000
Dataset 4	Metagenomic shotgun	Simulated	10	23,814,845	Simulated Illumina
