# Supplementary material for: Parallel-META 3: Comprehensive taxonomical and functional analysis platform for efficient comparison of microbial communities
Source: Sci Rep. 2017 Jan 12;7:40371. doi: 10.1038/srep40371 (PMC5227994; doi:10.1038/srep40371)
Supplement: Supplementary Information [file srep40371-s1.pdf]

# Supplementary file S1 for Parallel-META 3: Comprehensive taxonomical and functional analysis platform for efficient comparison of microbial communities

Gongchao Jing, Zheng Sun, Honglei Wang, Yanhai Gong, Shi Huang, Kang Ning, Jian Xu and Xiaoquan Su*

Single-Cell Center, Shandong Key Laboratory of Energy Genetics and CAS Key Laboratory of Biofuels, Qingdao Institute of Bioenergy and Bioprocess Technology, Chinese Academy of Sciences. Qingdao, Shandong, China, 266101

*suxq@qibebt.ac.cn

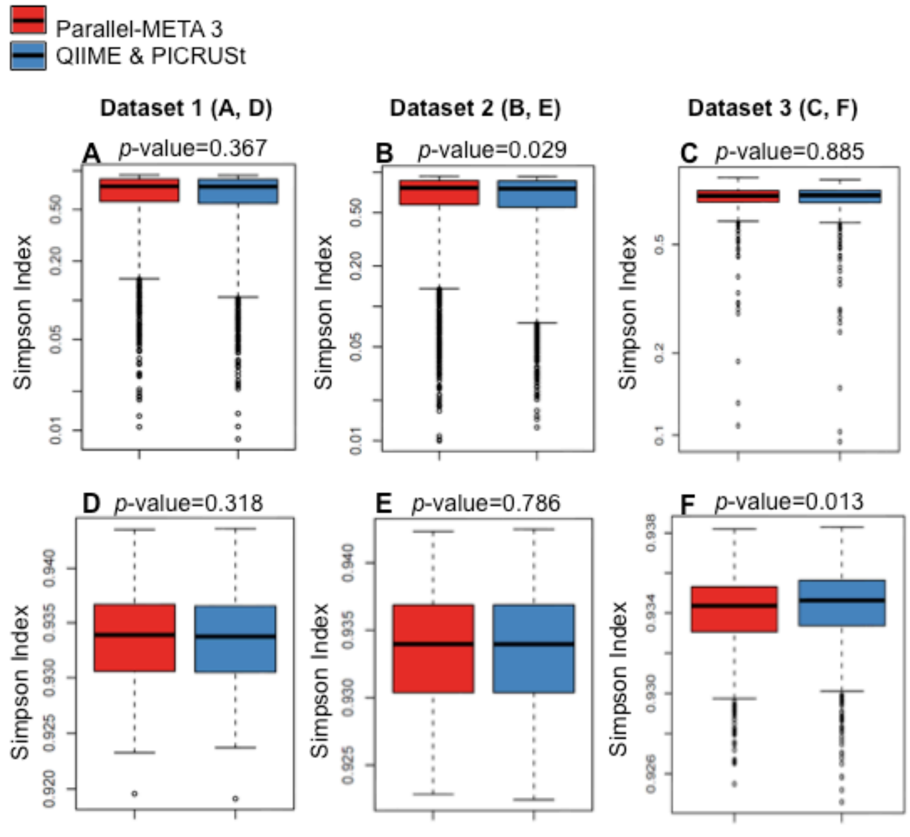


**Figure S1.** Simpson index of α diversity validated by QIIME for taxonomy structure on genus level (**A**: Dataset 1, **B**: Dataset 2 and **C**: Dataset 3) and by PICRUSt for predicted functional profiles of pathway (**D**: Dataset 1, **E**: Dataset 2 and **F**: Dataset 3).

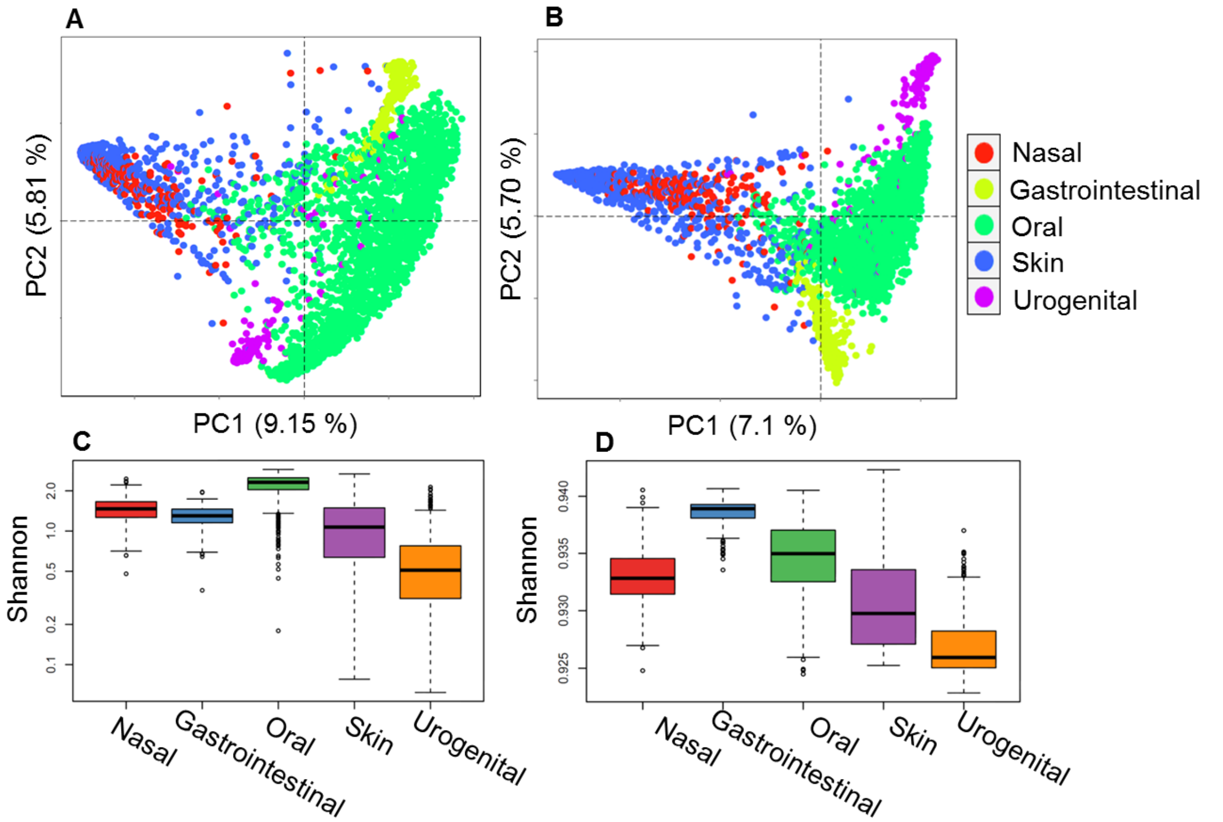


**Figure S2**. Community diversity variations among body sites of dataset 2. **A** and **B**: Partition of β diversity on taxonomy and functions of Dataset 2 based on Meta-Storms distances; **C** and **D**: Shannon index of α diversity of Dataset 2 on genus level and pathway.

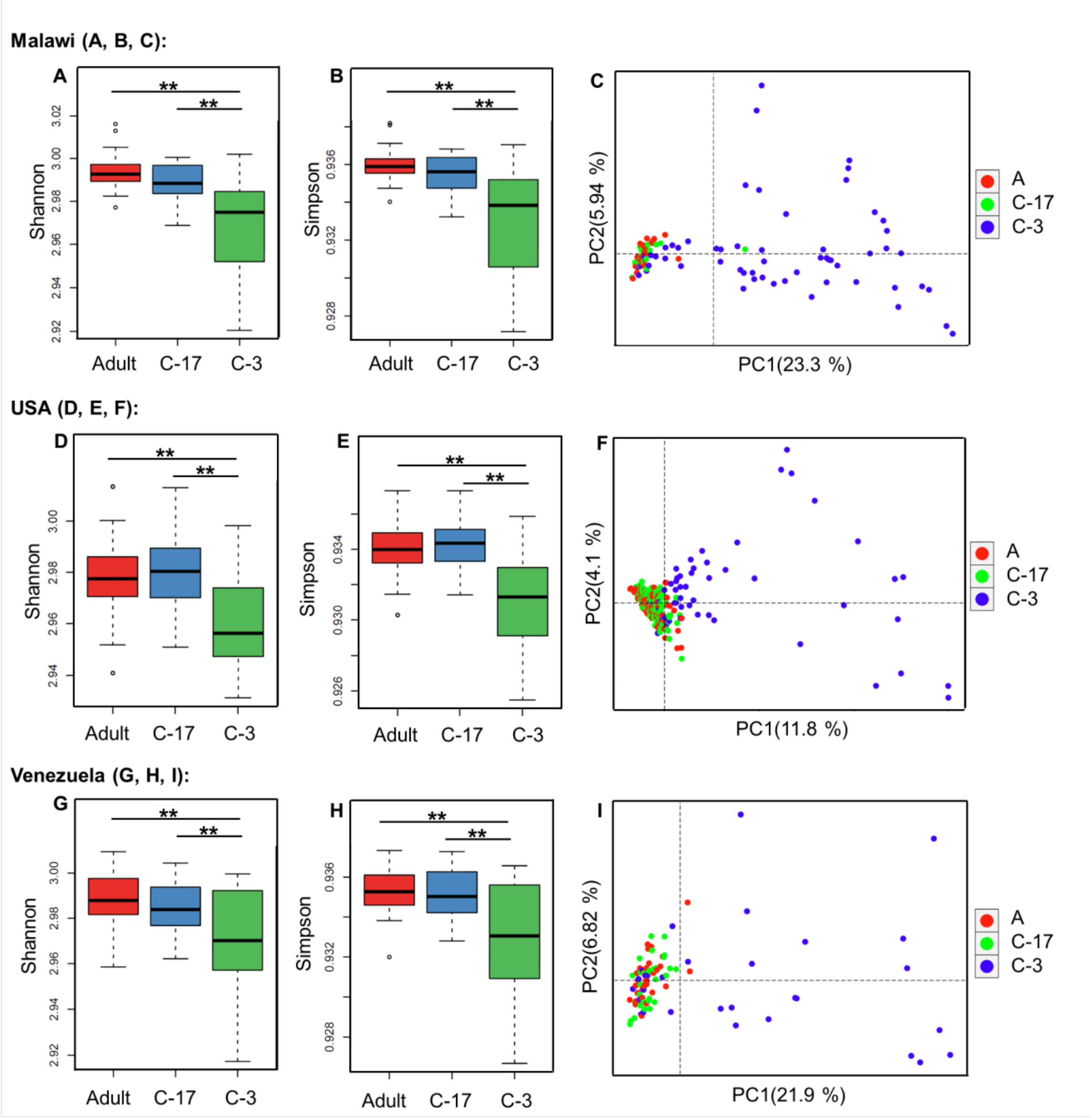


**Figure S3.** Functional diversity among samples of adults and children from 3 countries. α diversity was measured by Shannon index and Simpson index (**A** and **B** for Malawi, **D** and **E** for US, **G** and **H** for Venezuela); β diversity was illustrated in PCoA based on Meta-Storms distance (**C** for Malawi, **F** for US and **I** for Venezuela).

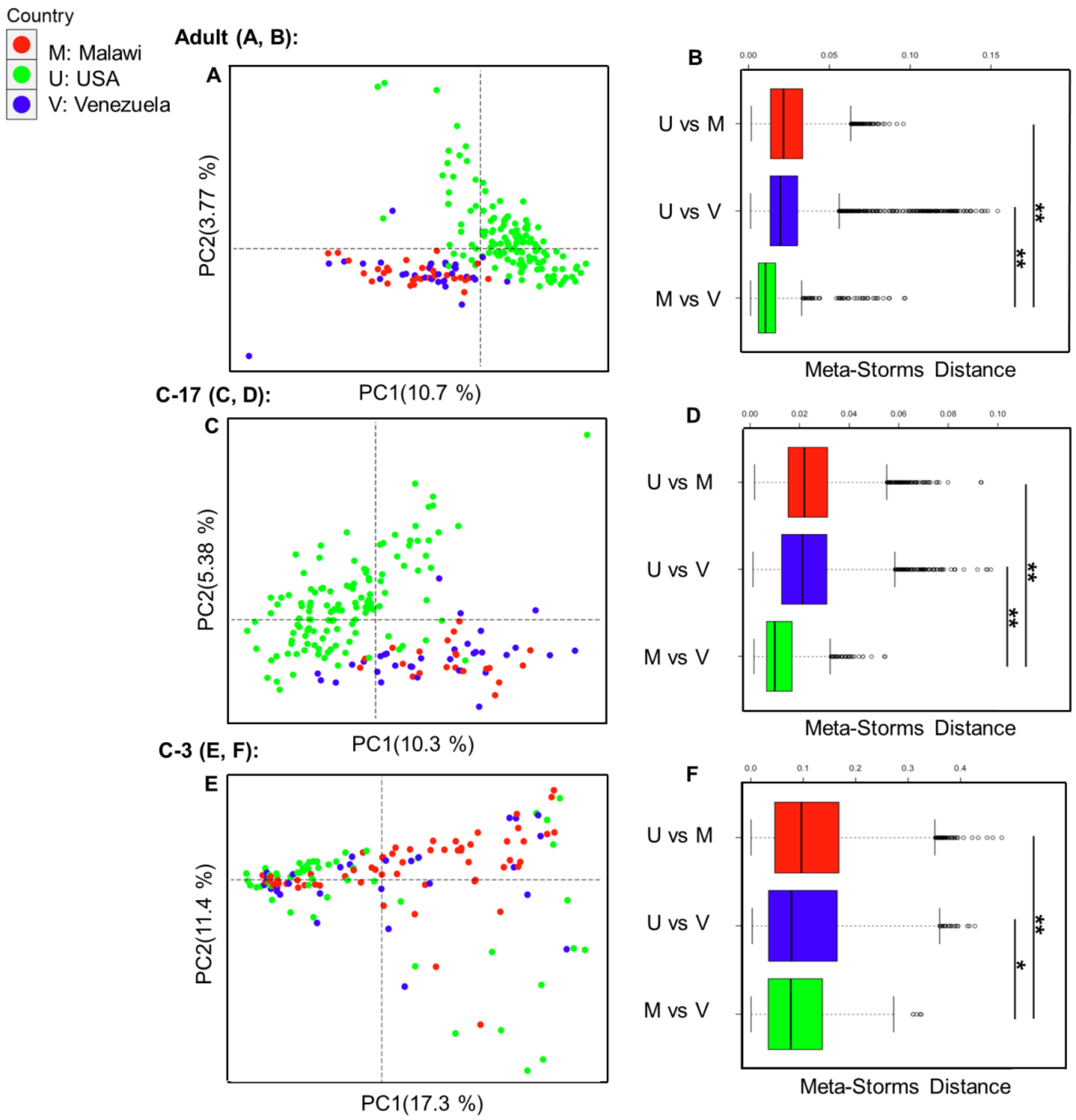


**Figure S4.** Significant functional variation of microbial community compositions were observed among gut samples from different countries of all age stages. Meta-Storms distances between Malawi samples and Venezuela samples were smaller than that between other pairs.

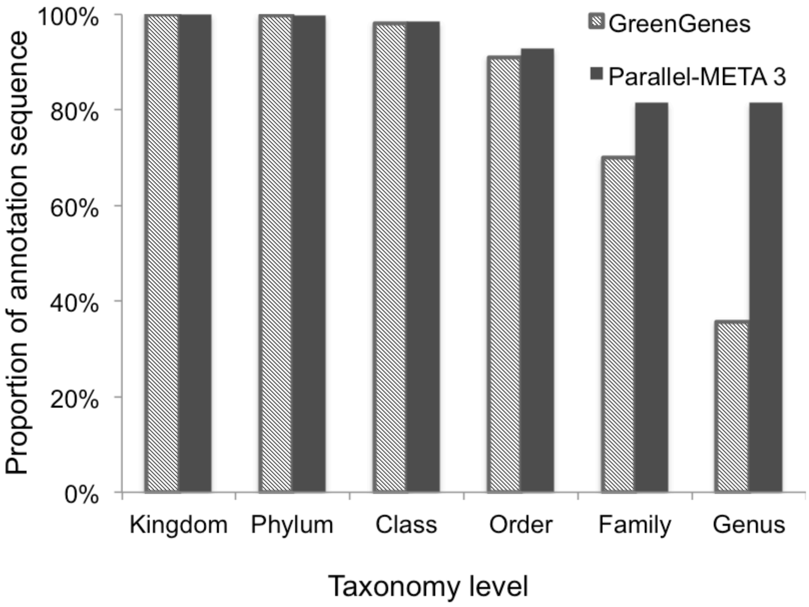


**Figure S5**. The integration of GreenGenes, RDP and SILVA improved the taxonomy annotation of Parallel-META 3 16S rRNA reference database. Reference sequences are from GreenGenes (version 13-8, sequence similarity on 97% level), and also integrated RDP and SILVA consensus taxonomy annotation (assigned by BLASTN with e-value < 1e-30 and similarity > 97%), which made the proportion of annotated sequences on genus level raised from 35.8% to 81.5% compared to the original GreenGenes annotation.

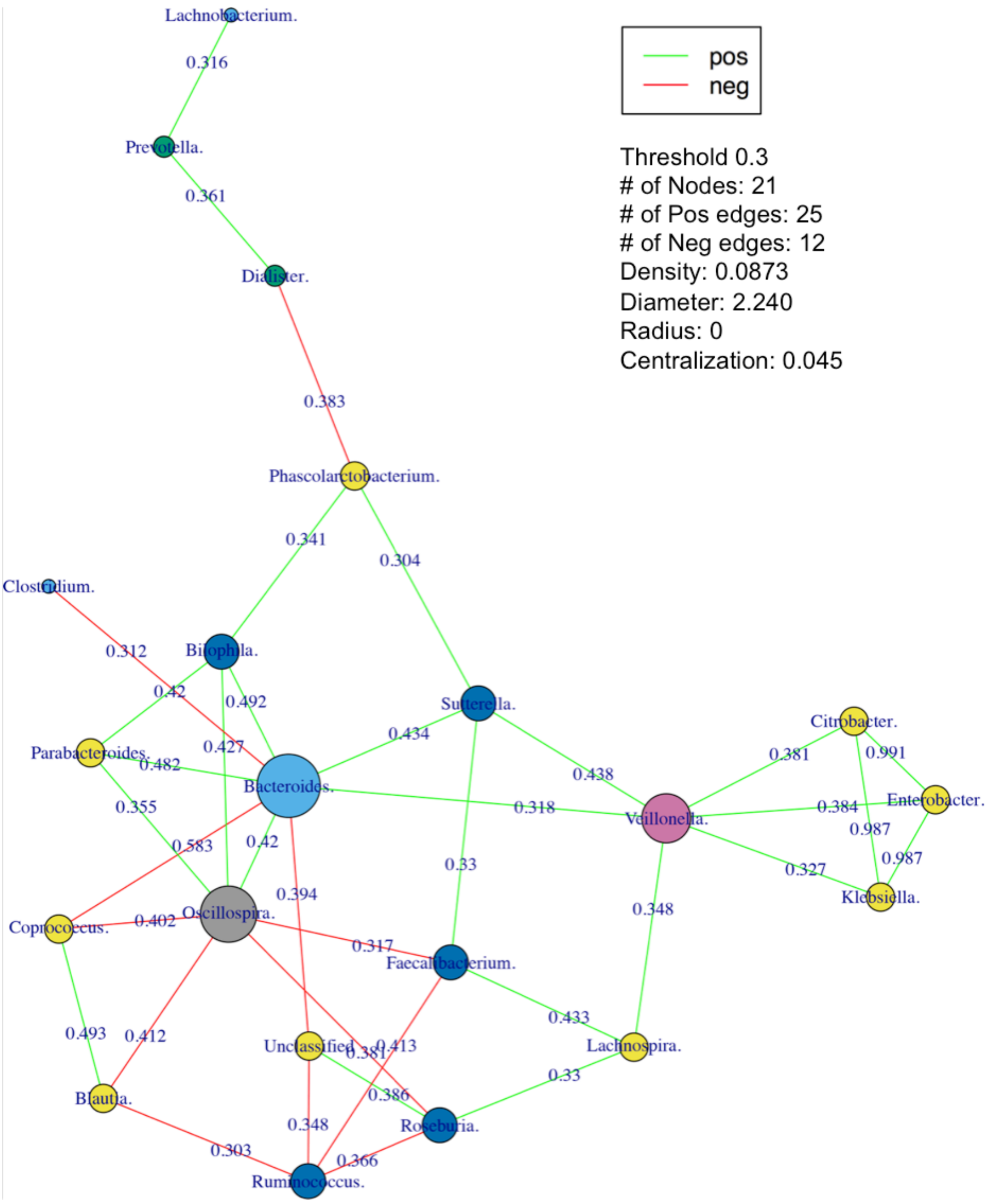


**Figure S6**. An example of the microbial interaction network. In the interaction network, each node is a single organism, and the sizes of nodes represent their proportions (abundances). Nodes are connected by edges that are calculated by their correlation coefficient of abundance variation among multiple samples. Edges in green color are positive correlation coefficient that means the co-occurrence, as well as red color edges means negative correlation coefficient that means the co-exclusion.

**Table S1.** Simulation design of metagenomic shotgun sequencing samples in dataset 4. 10 Metgenomeic samples were simulated with 9 genomes including *Actinomyces dentalis, Campylobacter gracilis, Fusobacterium nucleatum, Megasphaera micronuciformis, Neisseria canis, Porphyromonas endodontalis, Rothia aeria, Streptococcus parasanguinis* and *Veillonella parvula* downloaded from NCBI, short reads were produced by Dwgsim (version 0.1.8) in to Illunima 250 pb single-ended format with 50x coverage and 0.1% mutations. 10 Samples were divided into 2 groups by the different abundance distributions: samples in group 1 were dominated by *Actinomyces dentalis, Campylobacter gracilis* and *Fusobacterium nucleatum*, while samples in group 2 had high proportions of *Porphyromonas endodontalis, Rothia aeria* and *Streptococcus parasanguinis*.

| | **Sim_1** | **Sim_2** | **Sim_3** | **Sim_4** | **Sim_5** | **Sim_6** | **Sim_7** | **Sim_8** | **Sim_9** | **Sim_10** |
|---|---|---|---|---|---|---|---|---|---|---|
| *Actinomyces* | 33.33% | 40.01% | 33.34% | 43.34% | 26.67% | 6.67% | 6.67% | 6.66% | 6.67% | 6.67% |
| *Campylobacter* | 26.67% | 19.99% | 13.34% | 20.00% | 13.33% | 6.66% | 6.67% | 6.66% | 6.67% | 6.67% |
| *Fusobacterium* | 13.32% | 6.67% | 19.99% | 13.33% | 20.00% | 3.34% | 3.33% | 3.33% | 3.33% | 3.33% |
| *Megasphaera* | 6.67% | 13.34% | 13.34% | 6.66% | 13.33% | 3.33% | 3.33% | 3.34% | 3.33% | 3.33% |
| *Neisseria* | 6.67% | 6.67% | 6.66% | 6.67% | 13.33% | 6.67% | 13.33% | 6.66% | 13.33% | 13.33% |
| *Porphyromonas* | 3.33% | 6.66% | 6.67% | 3.33% | 3.33% | 13.33% | 6.67% | 20.00% | 13.34% | 19.99% |
| *Rothia* | 3.34% | 0.00% | 3.33% | 3.33% | 3.34% | 20.01% | 6.67% | 33.34% | 13.34% | 20.00% |
| *Streptococcus* | 3.34% | 3.33% | 0.00% | 3.33% | 3.33% | 26.66% | 33.33% | 13.34% | 26.66% | 20.00% |
| *Veillonella* | 3.34% | 3.33% | 3.33% | 0.00% | 3.33% | 13.33% | 20.00% | 6.67% | 13.33% | 6.67% |
| **Groups** | 1 | 1 | 1 | 1 | 1 | 2 | 2 | 2 | 2 | 2 |
| **# of reads** | 2,485,880 | 2,592,314 | 2,289,958 | 2,436,492 | 2,204,678 | 2,423,851 | 2,478,191 | 2,307,159 | 2,358,171 | 2,238,151 |
| **File size** | 1.3 GB | 1.4 GB | 1.2 GB | 1.3 GB | 1.2GB | 1.3 GB | 1.3GB | 1.2 GB | 1.3 GB | 1.2 GB |
